# Musculoskeletal Pain Is Associated with Dietary Diversity Score among Community-Dwelling Older Adult: A Cross-Sectional Study

**DOI:** 10.1155/2022/4228925

**Published:** 2022-02-07

**Authors:** Zahra Tajary, Zahra Esmaeily, Mahshid Rezaei, Shahrzad Daei, Atefeh Eyvazkhani, Marjan Mansouri Dara, Ahmad Reza Dorosty Motlagh

**Affiliations:** ^1^Department of Community Nutrition, School of Nutritional Sciences and Dietetics, Tehran University of Medical Sciences, Tehran, Iran; ^2^Department of Nutrition, Sciences and Research Branch, Islamic Azad University, Tehran, Iran

## Abstract

**Background:**

Musculoskeletal pains (MSP) are the most common cause of long-term severe pain and physical disability among older adults. This study is aimed at determining the relationship between dietary diversity score (DDS) and MSP in Tehran's older adults.

**Methods:**

The study was a cross-sectional one that employed 213 participants with and without MSP complaints between May and October 2019 in Tehran, Iran. A 100 mm length visual analog scale questionnaire was used to assess pain along with a validated 147-item food frequency questionnaire for DDS evaluation. Statistical analyses included descriptive analysis and multiple linear regression with a significance level of *p* < 0.05.

**Results:**

85% of the participants had a range of MSP with a low but insignificant DDS compared to individuals without pain (*p* = 0.12, 3.24 (±0.86) vs. 3.43 (±0.85), respectively). A significant association was observed among the quartiles of DDS that most of the subjects with MSP were in the lowest quartile relative to the highest one (*p* = 0.02). Moreover, the association between DDS and MSP remained significant in the adjusted model (OR = 0.28, 95%(CI) = 0.08 − 0.99).

**Conclusion:**

A high-quality diet is important. Our study showed that a higher dietary diversity might be associated with lower MSP in older adults. More robust interventional studies are thus warranted to confirm the results.

## 1. Introduction

Musculoskeletal disorders (MSD) are the second major cause of disabilities globally. They are characterized by the presentation of pain and physical dysfunction [1, 2]. MSDs include pain and movement restriction with a possible long-lasting impact. Osteoarthritis, rheumatoid arthritis, back pain, and neck pain are the most common MSDs. About 20-30% of the world population have a painful MSD [1]. Musculoskeletal pain (MSP) is a kind of MSDs which has many negative effects on people's health and comes at a great cost to society [2]. Unhealthy diet, alcohol consumption, smoking, lack of physical activity, frequent use of medication, and aging are reported as the common risk factors associated with the development of MSP [3]. However, no study has reported the association between dietary quality and MSP in Iran so far.

Dietary pattern quality is a way to reflect a diet status which has been assessed by a number of studies rather than using specific nutrients to investigate the relationship between dietary pattern and health status [4–6]. While studying a single nutrient alone may provide little information about the dietary status, a dietary pattern can determine the simultaneous effects of nutrients interacting with one another [7, 8]. Several indices for evaluating dietary pattern quality have been reported, and Dietary Diversity Score (DDS) remains one of them [7, 12]. DDS is a qualitative measurement of food consumption and represents a household's access to a variety of food [9].

Different studies have shown the association between MSP and adherence to a particular diet or nutrient intake [10–18]. Moreover, MSP was associated with body weight, minerals, and vitamins such as calcium and vitamin D in the studies of Iran and Minneapolis of the United States [10–12]. An exploratory cross-sectional study on 1424 adolescents at 14 years of age, in Western Australia, showed that low egg and high meat consumption were associated with a reduced risk of back and neck pain, respectively, in women. There was also a relationship between decreased vitamin B12 intake and increased risk of neck pain in women. Low and high consumption of cereals were also linked with an elevated risk of neck pain [13]. Yang and Haldeman suggested that there was a positive association between reported low back pain and obesity. On the other hand, the plant-based diet reduced MSP and improved participants' life quality [14]. Besides, consumption of plant-based foods impacted positively on chronic pain [15].

The association of DDS with the risk of various morbidities has been evaluated in former studies as well [16–18]. The prevalence of metabolic syndrome and risk of cardiovascular disease decreased with increased DDS in Iranian adults [16, 17]. Adherence to the DDS could also potentially prevent obesity and abdominal adiposity among female students [18].

To the best of our knowledge, there is currently a paucity of data on the association between DDS and the severity of MSP in older adults in Iran. This study thus is aimed at determining the positive association of DDS and sociodemographic factors as primary and secondary goals, respectively, with MSP lasting for at least the last three months in older residents of Tehran, Iran.

## 2. Materials and Method

### 2.1. Data Collection

A total of 213 older participants aged 60 years and above participated in this cross-sectional study between May and October 2019. Inclusion criteria for this study were MSP lasting for at least the last three months, no changes in the dietary habits during the year prior to the onset of MSP, no history of congestive heart failure, chronic obstructive pulmonary disease, chronic renal failure, cirrhosis, or active cancer, and no severe pains related to an accident, trauma, torsion, or fracture due to falls.

In order to evaluate the reliability of the questionnaires and sample size calculation, a pilot study was conducted on 100 older adults. Considering type I error of *α* = 0.05 and type II error of *β* = 80%, 126 participants with MSP and overall 158 older adults were needed for this study. Eventually, 213 subjects were studied to increase statistical power.

For sampling, the health care centers were divided into five regions firstly: North, South, East, West, and city center. Sampling was conducted in two stages. In the first stage, the healthcare centers of each region were listed, and then, several healthcare centers were selected after stratified sampling. In the second stage, the sampling was started from the first center until a sufficient number of patients were found. Written consent was obtained from participants after the rationale behind the study had been explained to them. General information was gathered by a standardized questionnaire including anthropometric measurements, level of physical activity, socioeconomic, and medical status. To estimate the physical activity level, the daily average time spent on exercising, jogging, or doing other sports was asked from subjects. To determine the socioeconomic status, we used a 9-item questionnaire (possession of house, car, side-by-side refrigerator, washing machine, dishwasher, laptop/personal computer, sofa, microwave, and handmade carpet) and also asked about possession of a personal house and car, and finally, we classified the economic status according to the following standards: very bad: ≤3 items without any personal home and car. Bad: ≤3 items with personal home or car. 4-6 items without personal home and car. Average: 4-6 items with personal home or car. 7 items ≤ without a personal home and car. Good: 7 ≤ items with personal home or car. Very good: 7 items ≤ with personal home and car [19].

This study was approved by the ethics committee of Tehran University of Medical Sciences with a protocol number of IR.TUMS.VCR.REC.1398.390.

### 2.2. Dietary Data Collection

We used a validated 147 item food frequency questionnaire (FFQ) in this study [20]. Main items included bread and grains, legumes, meat, and meat-derived products, poultry, fish, eggs, dairies, kinds of butter, vegetables, pickles, fruits and fruit juices, oils, seeds, and nuts, added sugar, drinks, spices, and salt. The consumption of each component in a day, week, month, or year together with the amount of consumption was asked by a trained dietitian.

### 2.3. Anthropometric Measurements

In our study, waist and hip circumferences, weight, and height were measured. For waist circumference measurement, the tape measure was placed about halfway between the bottom of the lowest rib and the top of hip bones, roughly in line with the belly button. Hip circumference was measured at the maximum width over the greater trochanters. Circumferences were measured with fiber-glass tape in the standing stage. Height (m) and weight (kg) were measured without wearing shoes on with light clothes by using a fiber-glass tape and Camry EB9011 scale (Camry Co, Zhongshan, China), respectively.

Body mass index (BMI) was calculated as the ratio of weight (kg) by the squared height (m). Waist to hip ratio (WHR) was calculated as waist circumference (cm) divided by hip circumference (cm), and waist to height ratio (WHtR) was the division of waist circumference (cm) to height (cm). WHtR higher than 0.6, waist circumference higher than 102 cm in men and 88 cm in women, was determined as abdominal obesity [21, 22]. WHR higher than 0.85 was assumed as an indicator of abdominal obesity in women as well, however, it was not used for men because of cultural and religious issues. Subjects with BMI < 23.5 were underweight and more than 30.9 were overweight/obese [23].

### 2.4. Calculation of Dietary Diversity Score

DDS calculation was based on Haines et al. suggestion [24]. Foods were classified into four main categories of meats and dairy, grains, fruits, and vegetables. Then, each one of meat and dairy, grains, fruits, and vegetables were divided into subgroups. Bread and cereals and its products include 7 subgroups (white bread, biscuits, pasta, whole-grain bread, whole grains, rice, and flours), vegetables include 7 subgroups (tomatoes and its products, vegetables Starch, legumes, yellow and orange vegetables, green leafy vegetables, and other vegetables such as eggplant and squash), fruits include 2 subgroups (the first group includes citrus fruits, summer vegetables, and berries, and the second group includes other fruits and fruit juice that include apples, bananas, grapes, raisins, etc.), and meats and dairy products also include 7 subgroups (milk, cheese, yogurt, red meats, chicken, poultry, eggs, fish, etc.). Eventually, 23 subgroups were formed. The maximum score of DD was 10, and each of the 4 categories was given a maximum score of 2.5. The scores of the 4 main groups were summed up to constitute the total score. To be considered as a “consumer” for any of the food categories, subjects should consume at least one-half serving of every subgroups' food list. For example, in the fruit group, if a person consumed half of a serving of citrus, the score was calculated as (1 ÷ 2) × 2.5 = 1.25, so that, the diversity score of the fruit group is 1.25 [24].

### 2.5. Musculoskeletal Pain

The participants were asked to subjectively indicate feeling the pain that exists every day for at least three months in any part of the hand, wrist, shoulder elbow, face, jaw, neck, thigh, knee, ankle, legs, and lower back during the past 6 months. Chronic MSP was assessed by using the visual analog scale (VAS) questionnaire. The VAS had the terms “no pain (zero)” and “most severe pain (10)” anchored at its ends from the left to the right [10]. We asked participants to mark a place on a scale that aligns with their level of pain. Then, we measured the line with a ruler to get a pain score [25].

### 2.6. Statistical Method

All statistical analyses were performed using IBM SPSS Software, Version 16. Initially, the normality of the variables was assessed by the Kolmogorov-Smirnov test. DDS was divided into quartiles to assess the relationship between DDS and pain. To evaluate the association between MSP and quantitative variables, the Student's *T*-test and analysis of variance (ANOVA) were used and the associations between quartiles of DDS and the severity of the pain were assessed by the *X*^2^ test. Pearson's correlation coefficient tested the relation between mean pain and quantitative variables. The multiple regression model was applied for controlling the confounders of the relationship between DDS and MSP. The level of significance was set at *p* < 0.05 in all analyses and was tested 1-sided.

## 3. Result

### 3.1. Characteristics of Study Participants

Demographic characteristics were summarized in Table 1. A total of 213 participants enrolled in the study that 23% (49) were men and 77% (164) were women which men experienced less MSP than women (*p* < 0.0001). The mean age of participants was 66.28 (±5.75) years. People with gastrointestinal, cardiovascular, pulmonary, or skeletal diseases had significantly high MSP scores (*p* = 0.02, *p* = 0.01, *p* = 0.05, *p* < 0.0001, respectively). Moreover, those who took gastrointestinal, psychiatric, or skeletal medications were affected by pain. It was also found that people with MSP had higher WHtR compared to the other group. The most-reported scores were 5 (19.7%), 0 (15%), and 10 (12.7%) in this population, respectively.

### 3.2. Correlation between Pain Score and Continuous Variables

This study indicates significant positive correlations between pain and WC (*r* = 0.164, *p* = 0.008), BMI (*r* = 0.21, *p* = 0.001), WHtR (*r* = 0.23, *p* < 0.0001), WHR (*r* = 0.14, *p* = 0.04), hip circumference (*r* = 0.16, *p* = 0.02), number of pregnancies (*r* = 0.14, *p* = 0.04), and number of childbirth (*r* = 0.15, *p* = 0.03). Height had a significant inverse correlation with pain (*r* = −0.11, *p* = 0.05). This association demonstrated that those who were short had more pain.

### 3.3. Dietary Diversity and Pain Severity

Mean DDS in people with and without pain were 3.24(±0.86) and 3.43(±0.85), respectively. Nevertheless, no significant differences were observed between them. Furthermore, a high proportion of those feeling pain was in the lowest quartile in relation to the highest one (*p* = 0.02) (Table 2).

### 3.4. MSP, DDS, and Confounders

A significant negative association was found between MSP and DDS in both unadjusted (*B* = −0.16, *p* = 0.09) and adjusted (*B* = −0.13, *p* = 0.1) models. 21% of modifications were relevant to covariates in regards to the association between DDS and MSP (Table 3). Considering Table 4, those in the top DDS quartile were 72% less likely to have MSP in the adjusted model versus those in the lowest quartile.

## 4. Discussion

For all we know, this is the first study attempt to investigate the association between DDS and MSP. The result of this study indicated a possible protective role of DDS in MSP. Limited studies have been established on the association between dietary patterns and MSP, however, multiple studies assessed the role of nutrient intake in MSP occurrence [10, 12, 26, 27]. The impact of vitamin D intake on MSP has been widely considered which 25-hydroxyvitamin D deficiency has been reported to be associated with accelerated pain in MSP specifically in South Asian populations [26]. As Abbasi et al. stated, reduced risk of MSP was associated with a high intake of vitamin D [10]. A study by Heidari et al. showed a positive association between low levels of vitamin D and types of nonspecific bone pain, particularly in women [12]. Other similar studies represented a linkage between the abovementioned factors, however, there are a few studies that indicated no relationship between MSP and low levels of vitamin D [27]. Also, various studies have focused on the relationship between dietary components and MSP. Høstmark et al. suggested that eating fruits/berries and fruit juices together with balanced fatty acid patterns may improve MSP [28]. The result of the Towery et al. study showed that consumption of a plant-based diet rich in fruits, vegetables, and whole grains could have positive effects on MSP amelioration [15].

A few studies have examined the association between dietary patterns and types of pain. The lack of protein intake increases pain in patients with fibromyalgia by the suggestion of Barista et al. [29]. Another study pointed out that rapid and effective weight loss through a calorie-restricted diet with very few side effects could significantly improve symptoms in overweight patients with knee osteoarthritis [30]. No significant relationship was seen between spinal pain and diet quality through Perry et al.'s study [13]. It was remarked that DDS is a good indicator of diet quality, and our study has shown the correlation with MSP. A greater DDS was associated with less pain here. Oxidative stress is linked with inflammatory pain which could intensify proinflammatory peptides secretion with the assistance of leukocytes and neuroinflammatory processes. Accordingly, foods rich in antioxidants like fruits and berries might reduce pain by modulating the redox reactions and function of various enzymes. Hence, a diet low in antioxidants content may exacerbate oxidative stress in the body. On the other hand, increasing the omega-3/omega-6 fatty acids ratio can suppress the production of inflammatory cytokines, which in turn may reduce pain [28]. Human studies proposed that a diet rich in vegetables, fruits, healthy oils, and fiber has potent anti-inflammatory activity. The Mediterranean diet, which is well supplied in vegetables, fruits, fish, legumes, olive oil, and vegetable proteins, reduces chronic inflammation by downregulating C-reactive protein and interleukin-6 levels. In contrast, the consumption of (red) meat, sugar, bread, sweets, processed grains, white rice, white potatoes, and fried foods has a proinflammatory effect [31].

In the present study, higher DDS was associated with lower pain, which might be by the beneficial effects of dietary diversity components on inflammation and ultimately MSP as mentioned before. A healthier lifestyle might be the justification of the relationship between higher DDS and less pain.

Considering the sociodemographic factors, several studies showed a direct association between psychological disease and pain in line with our findings [32, 33]. Being a housewife in the present study was associated with increased pain. This may be due to having less opportunity to relax or exercise. Some factors such as physical activity, mental stress, and a sedentary lifestyle can be considered as predisposing factors for MSP in housewives. A study by Habib et al. showed that the number of hours worked at home, the number of children at home, fatigue by working at home, stress, and repetitive movements in addition to inappropriate positions while working at home were associated with MSP [34].

In this study, MSP was more common in women than men. As stated by Winkleby et al., sex-related differences in MSP were on account of sex hormones differences. On this matter, estrogen and/or progesterone are the main female sex hormones that may play a protective role in MSP [35].

Our study also suggested that people with university education had less pain in comparison with nonuniversity educated people. Inversely, numerous studies have shown that higher education is associated with more health problems, functional limitations, and disabilities [12, 36, 37].

Unexpectedly, severe pain was reported by those who took gastrointestinal, psychiatric, or skeletal medications than those who did not in the current study which additional studies were required to warrant confirmation.

This study has several strengths. First and foremost, samples were collected from five regions: north, south, east, west, and city center of Tehran as we tried to take into account the differences in the socioeconomic status of individuals. This study was conducted in Iran and even in the world for the first time to identify the association between DDS and MSP. Our study had some limitations as well. Primarily, this is a cross-sectional study without representing the cause-effect relationship. Another possible limitation is the under or overreporting of food intake by individuals. The use of FFQ is also a limitation. Not only do FFQ reflect the long-term intakes but also they have a low correlation with real diet and could have more measurement errors [38].

## 5. Conclusion

In conclusion, a dietary pattern with a greater diversity might have a protective effect on MSP among older adults. High-educated people may also experience less pain. Conversely, those taking gastrointestinal, psychiatric, or skeletal medications may suffer from severe MSP, plus, women might feel more pain than men. This finding warrants confirmation in high-quality interventional studies.

## Figures and Tables

**(a):** 

Variables (mean ± SD)		Musculoskeletal statues			*p* value∗∗
	With pain (*n* = 181) Mean	SD	Without pain (*n* = 32) Mean	SD	
Age (year)	66.75	5.74	66.20	5.76	0.31
Weight (kg)	72.98	11.63	74.67	8.71	0.22
Height (cm)	158.22	8.09	162.47	9.25	<0.0001
Body mass index (kg/m^2^)	29.16	4.21	28.43	3.76	0.18
Family number	5.43	1.78	4.77	1.28	0.03
Sleep duration (hour)	6.92	1.90	7.19	1.44	0.23
Physical activity	34.90	41.08	39.63	28.26	0.27
Waist to height ratio (WHtR)	0.61	0.07	0.6	0.06	0.05
Menstruation age (y)∗	13.13	1.8	13	1.78	0.39
Postmenopausal (y)∗	47.58	5.51	47.12	5.51	0.37
Hip circumference (cm)∗	110.22	9.06	111.75	6.67	0.25
Waist to hip ratio (WHR)∗	0.88	0.07	0.85	0.07	0.10

**(b):** 

Variable *N* (%)	Total (*n* = 213)	With pain (*n* = 181)	Without pain (*n* = 32)	*p* value∗∗∗
Gender				
Male	49	35 (71.4)	14 (28.6)	<0.0001
Female	164	146 (89)	18 (11)	
Marital statues				
Married	155	129 (83.2)	26 (16.8)	0.17
Other	58	52 (89.7)	6 (10.3)	
Protector				
Father	48	44 (91.7)	4 (8.3)	0.09
Mother	158	130 (82.3)	28 (17.7)	
Education				
High school or lower	151	134 (88.7)	17 (11.3)	0.02
University	62	47 (75.8)	15 (24.2)	
Spouse education				
High school or lower	136	119 (87.5)	17 (12.5)	0.15
University	74	60 (81.1)	14 (18.9)	
Former job				
Clerk	66	54 (81.8)	12 (18.2)	0.18
Housewife	104	92 (88.5)	12 (11.5)	
Other	25	20 (80)	5 (20)	
Latter job				
Housewife	114	103 (90.4)	11 (9.6)	0.03
Retired	78	63 (80.8)	15 (19.2)	
Other	10	7 (70)	3 (30)	
Disorders				
Gastrointestinal	71	66 (93)	5 (7)	0.02
Diabetes	52	44 (84.6)	8 (15.4)	0.55
Cardiovascular	63	59 (93.7)	4 (6.3)	0.01
Pulmonary	118	105 (89)	13 (11)	0.05
Renal	20	18 (90)	2 (10)	0.75
Cancer	21	19 (90.5)	2 (9.5)	0.36
Skeletal	142	135 (95.1)	7 (4.9)	<0.0001
Psychological	1	1 (100)	0 (0)	0.85
Medication				
Gastrointestinal	54	51 (94.4)	3 (5.6)	0.02
Diabetes	45	37 (82.2)	8 (17.8)	0.35
Cardiovascular	28	28 (100)	0 (0)	0.01
Skeletal	100	87 (87)	13 (13)	0.28
Psychological	57	56 (98.2)	1 (1.8)	<0.0001
Other	72	63 (87.5)	9 (12.5)	0.29
Supplements				
Vitamin D	147	127 (86.4)	20 (13.6)	0.25
Multivitamins	80	68 (85)	12 (15)	0.58
Minerals	106	90 (84.9)	16 (15.1)	0.51
Economic stat				
Very bad	9	8 (88.9)	1 (11.1)	0.17
Bad	31	28 (90.3)	3 (9.7)	
Moderate	81	71 (87.7)	10 (12.3)	
Good	19	17 (89.5)	2 (10.5)	
Very good	72	56 (77.8)	16 (22.2)	

∗In females. ∗∗*p* ≤ 0.05; independent sample *T*-test. ∗∗∗*p* ≤ 0.05; *X*^2^ test used for distribution of qualitative variables. WHtR categories: normal, WHtR < 0.6; abdominal obesity, WHtR ≥ 0.6. WHR categories: normal, WHR < 0.85; abdominal obesity, WHR ≥ 0.85. BMI categories: underweight, BMI < 23; normal, 23 < BMI < 30; obese > 30.

**Table 2:** Association of pain severity and dietary diversity.

Variable		With pain (*n* = 181)	Without pain (*n* = 32)	*p* value∗
Dietary diversity score (Mean ± SD)		3.24 ± 0.86	3.43 ± 0.85	0.12

Diet diversity score, *N* (%)	Q1 (1.43-2.50)	54 (91.5)	5 (8.5)	0.02
	Q2 (2.51-3.21)	40 (75.5)	13 (24.5)	
	Q3 (3.22-3.93)	46 (92)	4 (8)	
	Q4 (3.94-5.54)	41 (80.4)	10 (19.6)	

Q: quartiles. ∗*p* ≤ 0.05; Student's *T*-test used for comparing mean of quantitative variables and *X*^2^ test used for distribution of qualitative variables.

**Table 3:** Multiple linear regression for MSP and DDS.

Variables	Adjusted *R*^2^	Unstandardized coefficients	CI (95%)	*p* value∗∗∗
		B (SE)		
Crude model	0.004	-0.16 (0.12)	(-0.41-0.08)	0.09
Model I∗	0.05	-0.15 (0.12)	(-0.39-0.09)	0.1
Model II∗∗	0.21	-0.13 (0.11)	(-0.35-0.1)	0.1

∗Adjusted for gender. ∗∗Adjusted for gender, age, economic status, history of CVD, skeletal disorders, skeletal medications, and BMI. ∗∗∗*p* ≤ 0.1. MSP: musculoskeletal pain; DDS: dietary diversity score.

**Table 4:** Logistic regression: musculoskeletal pain.

Variables	Crude model	Model I∗	Model II∗∗
	OR 95% (CI)	OR 95% (CI)	OR 95% (CI)
Dietary diversity score model			
Quartile 1	1.00	1.00	1.00
Quartile 2	0.29 (0.09-0.86)	0.3 (0.09-0.93)	0.41 (0.12-1.46)
Quartile 3	1.07 (0.27-4.2)	1.09 (0.27-4.4)	1.67 (0.37-7.57)
Quartile 4	0.38 (0.12-1.2)	0.37 (0.11-1.19)	0.28 (0.08-0.99)
*p* trend	< 0.0001	0.35	0.13

∗Adjusted for gender. ∗∗Adjusted for gender, age, economic status, history of CVD, skeletal disorders, skeletal medications, and BMI.

## Data Availability

The data used to support the findings of this study are available from the corresponding author upon request.
